# SPAED: harnessing AlphaFold output for accurate segmentation of phage endolysin domains

**DOI:** 10.1093/bioinformatics/btaf531

**Published:** 2025-09-24

**Authors:** Alexandre Boulay, Emma Cremelie, Clovis Galiez, Yves Briers, Elsa Rousseau, Roberto Vázquez

**Affiliations:** Department of Biotechnology, Ghent University, Ghent B-9000, Belgium; Département de biochimie, de microbiologie et de bio-informatique, Université Laval, Québec, QC, G1V 0A6, Canada; Centre Nutrition, Santé et Société (NUTRISS), Institute of Nutrition and Functional Foods (INAF), Université Laval, Québec, QC, G1V 0A6, Canada; Department of Biotechnology, Ghent University, Ghent B-9000, Belgium; Univ. Grenoble Alpes, CNRS, Grenoble INP, LJK, Grenoble 38000, France; Department of Biotechnology, Ghent University, Ghent B-9000, Belgium; Centre Nutrition, Santé et Société (NUTRISS), Institute of Nutrition and Functional Foods (INAF), Université Laval, Québec, QC, G1V 0A6, Canada; Département d’informatique et de génie logiciel, Université Laval, Québec, QC, G1V 0A6, Canada; Centre de Recherche en Données Massives (CRDM), Université Laval, Québec, QC, G1V OA6, Canada; Institut Intelligence et Données (IID), Université Laval, Québec, QC, G1V 0A6, Canada; Department of Biotechnology, Ghent University, Ghent B-9000, Belgium; Centro de Investigación Biomédica en Red de Enfermedades Respiratorias (CIBERES), Madrid 28029, Spain

## Abstract

**Summary:**

SPAED is an accessible tool for the accurate segmentation of protein domains that leverages information contained in the predicted aligned error (PAE) matrix obtained from AlphaFold to better identify domain-linker boundaries and detect terminal disordered regions. On a dataset of 376 bacteriophage endolysins (proteins that degrade the bacterial cell wall), SPAED achieves a mean intersect-over-union score of 96% and a domain-boundary-distance score of 89% compared to 94% and 70%, respectively, for the state-of-the-art tool Chainsaw.

**Availability and implementation:**

Implemented in Python, SPAED is accessible on the web (https://spaed.ca) and available for download from https://github.com/Rousseau-Team/spaed or https://pypi.org/project/spaed. The data used to test SPAED can be found at https://doi.org/10.5281/zenodo.15285860.

## 1 Introduction

Bacteriophages (phages), viruses that infect bacteria, are some of the most abundant and diverse biological entities on Earth (Labrie *et al.* 2010, Dion *et al.* 2020). To release their progeny into the environment, phages often rely on endolysins that degrade the peptidoglycan layer of the bacterial cell wall thus enabling lysis of the host (Oliveira *et al.* 2013, Cahill and Young 2019). The complexity and variety of bacterial cell wall architectures have driven phages to refine their lytic cassette to be tailored to their host (Oechslin *et al.* 2022). Consequently, the diversity of endolysins mirrors that of the phages themselves and of their hosts (Criel *et al.* 2021, Vázquez *et al.* 2021).

Many phage endolysins have a modular structure, with each module usually possessing either a cell wall-binding or catalytic function (Schmelcher *et al.* 2012, Vázquez *et al.* 2021). This modularity facilitates the acquisition of new domains—through recombination in nature or domain engineering in the lab (Schmelcher *et al.* 2012, Gerstmans *et al.* 2020, Oechslin *et al.* 2022). The accurate identification of lysin domains is thus important from a biological perspective as well as for the development of new antimicrobial agents (Schmelcher *et al.* 2012, Gerstmans *et al.* 2018).

Existing tools for protein domain segmentation are not specifically adapted to endolysins. Since the advent of AlphaFold2/3 (Jumper *et al.* 2021, Abramson *et al.* 2024), many state-of-the-art tools use supervised deep learning models trained on large datasets and based on structural information (Eguchi and Huang 2020, Lau *et al.* 2023, Wells *et al.* 2024). They are shown to work well in general but depend on the quality of annotations present in these databases. Although they have grown in recent years, protein domain databases are not necessarily representative of all modular proteins, and phage proteins are particularly underrepresented (Wang *et al.* 2021, Lee *et al.* 2024). In contrast to supervised approaches, unsupervised-heuristic algorithms have also been used historically, but these approaches typically struggle to encompass all cases (Redfern *et al.* 2007, Zhang *et al.* 2023).

Here we developed SPAED, a tool for the **S**egmentation of **P**h**A**ge **E**ndolysin **D**omains that applies hierarchical clustering to the predicted aligned error (**PAE**) matrix obtained from AlphaFold predictions. The PAE is a score that estimates the expected positional error for each pair of residues in a predicted protein structure by calculating the error associated with aligning each residue to every other (Guo *et al.* 2022). It is a measure of the local packing of residues and relative placement of domains in a protein. SPAED uses these expected positional errors as a measure of how likely residues are to be found in the same domain. This approach is well suited for endolysins because their domains are mostly compact and separate from one another, which is reflected in the PAE matrices of these proteins. SPAED was tested extensively on a dataset of 376 manually delineated endolysins and we also demonstrate its applicability to other types of modular proteins obtained from CASP12 (Moult *et al.* 2018). SPAED can easily be launched from our web portal available at www.spaed.ca and is downloadable through GitHub and PyPI for ease of use on larger datasets.

## 2 Methods

A dataset of 376 endolysins was obtained from previous and ongoing projects performed at Ghent University (Criel *et al.* 2021, Vázquez *et al.* 2024, 2025) (https://doi.org/10.5281/zenodo.15285860). The 3D structures of all lysins were predicted using ColabFold v1.5.5 (Mirdita *et al.* 2022) and the PAE files were collected. The ground truth (GT) delineations, serving as benchmark, were obtained by visually identifying the domains in these predicted 3D structures using SwissPdb Viewer (Guex and Peitsch 1997). Domains are defined as compact, autonomously folded regions within the 3D structure and are delimited by the residue immediately adjacent to the secondary structures found at the boundaries of each compact region. Linkers are the less compact regions connecting two domains. They are usually short (5–15 residues), but can be longer and possess small elements of secondary structure. Terminal disordered regions are less compact regions found at either end of the protein and potentially correspond to signal peptides of interest (usually at least 25 residues long) (São-José *et al.* 2000, Nakonieczna *et al.* 2024). Importantly, this delineation process has been used in experimental work and has been shown to preserve the autonomous function of domains (Vázquez *et al.* 2024, 2025).

### 2.1 Algorithm

A complete example of the algorithm with explanations and visuals for each step is shown in Appendix 1, available as supplementary data at *Bioinformatics* online.

**Figure 1.**
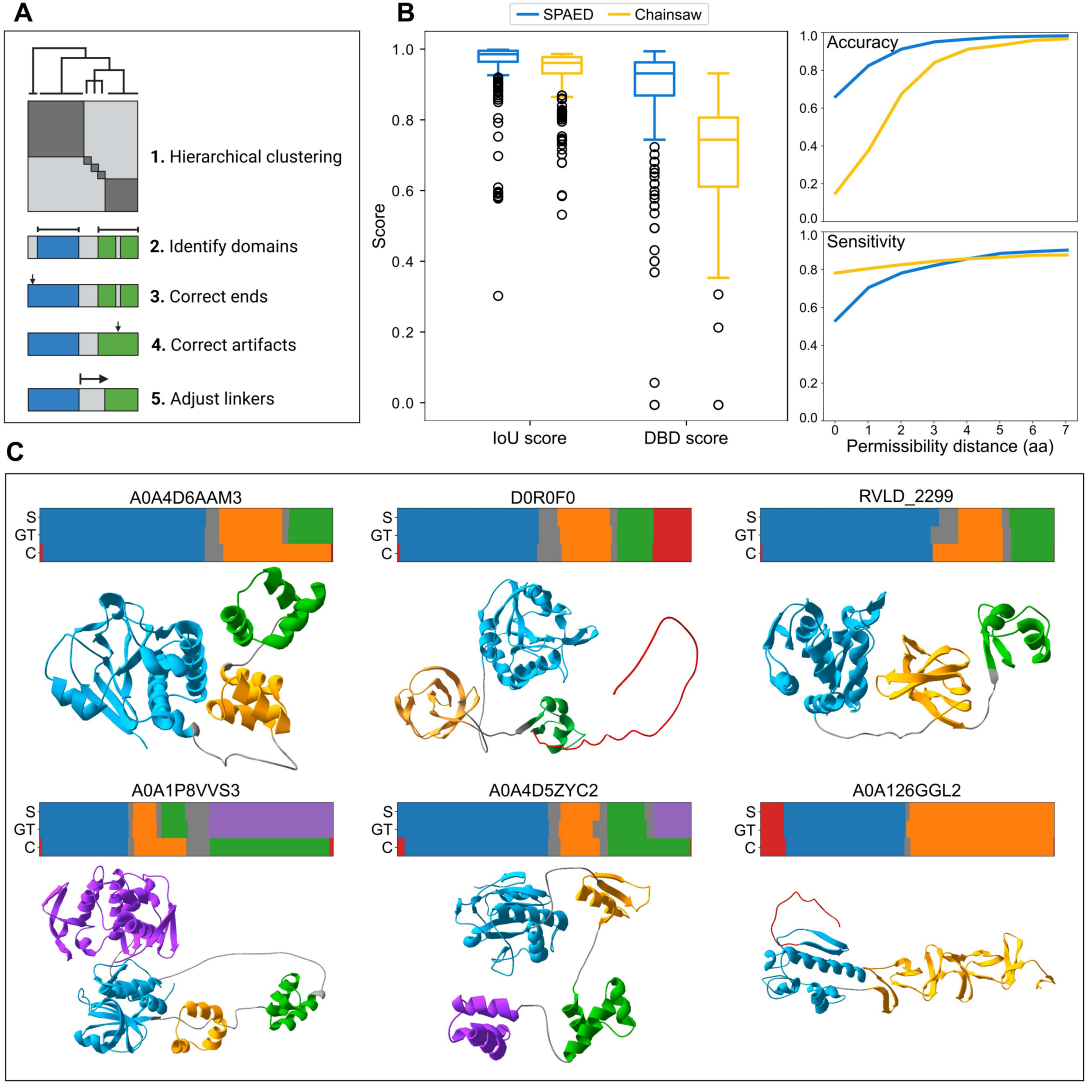
SPAED overview, performance evaluation and example delineations. (A) SPAED algorithm overview. (B) SPAED and Chainsaw’s (Wells 2024) performance on the 376 lysin dataset. Accuracy and sensitivity of boundary predictions were calculated over a permissibility range that allows for distances of 0–7 amino acids (aa) between the predicted and ground truth (GT) boundary when classifying a prediction as correct/incorrect (i.e. considering that predictions with distance ≤X to GT are correct). (C) Comparison of SPAED (S), ground truth (GT), and Chainsaw (C) domain segmentations for six endolysins representative of different architectures. 3D structures show domains as predicted by SPAED and were made using SwissPdb Viewer (Guex and Peitsch1997). Terminal disordered regions are shown in red, linkers in grey, and domains in other colors. Created in BioRender. Boulay, A. (2025) https://BioRender.com/b8ins4b. IoU: intersect over union; DBD: domain boundary distance.

*Step 1: Hierarchical clustering.* At the basis of SPAED is a single linkage hierarchical clustering algorithm (hierarchy.fclusterdata from SciPy) that takes as input a symmetrized PAE matrix from AlphaFold (Fig. 1A; step 1). This symmetrized matrix is obtained by averaging the PAE matrix and its transpose: $(\mathrm{pae}+{\mathrm{pae}}^{T})/2$. Applying the clustering to the columns of the resulting matrix places residues with similar profiles in the PAE matrix into the same cluster.

The hierarchical clustering is restricted to a maximum number of clusters (criterion = “maxclust”) that we set to 1/10th of the length of the protein after a series of tests (see performance_eval.ipynb in the GitHub repository), typically resulting in 20–60 clusters. This number is high compared to the expected number of domains as lysins are known to possess 1–4 domains. However, this allows a necessary flexibility to the clustering algorithm which then assigns a high number of small, preliminary clusters in less compact regions, such as the extremities of the protein and between domains (i.e. linker regions). As a result, many small, often-singleton clusters are produced in those regions, whereas long, structured clusters are generated in more compact regions.

*Step 2: Identify domains.* Clusters containing >25 residues are assigned as predicted domains (Fig. 1A; step 2). This number accounts for the smallest expected size of a domain (>30 residues) (Lin and Zewail 2012) and leaves a buffer for errors in the preliminary assignment of clusters. All other clusters (containing <25 residues) are assigned a “non-domain” identifier. According to their position, these “non-domain” residues can be (i) terminal disordered regions (see step 3) when they are found at either end of the protein, (ii) wrong assignments (see step 4) when they are found within a single domain, or (iii) linkers (see step 5) when they are found between two domains.

*Step 3: Correct ends.* Terminal regions labeled as “non-domain” are concatenated to the nearest domain if they are <20 residues long (Fig. 1A; step 3). Alternatively, an additional filter is applied to evaluate if the region is truly disordered by measuring the compactness of residues in the region. Put simply, if every residue in a region is only close in space to the residues that surround it in the amino acid sequence, the region is likely disordered (lacks tertiary structure). The compactness of each residue is measured by counting the number of neighboring residues in the region that have a “low” PAE score (<5). The latter threshold was defined through experimental observation on the lysin dataset (see Fig. A1.3, available as supplementary data at *Bioinformatics* online) and has been used before as a measure in other tasks (Watson *et al.* 2023). Then, if >80% of residues in the region have 5 neighbors or less with low scores, the region is considered disordered (not compact). If the terminal region is deemed to be compact, it is concatenated to the nearest domain as it likely corresponds to a less packed (but still ordered) region of that domain.

*Step 4: Correct artifacts.* Small errors that originate from the original clustering are then removed (Fig. 1A; step 4). For this, a simple sliding window is used to verify that all domains are continuous, making the necessary adjustments if that is not the case.

*Step 5: Adjust linkers.* Finally, linker boundaries are adjusted (Fig. 1A; step 5). Similarly to how terminal disordered regions are detected, residues that are part of a linker will have a low PAE score (PAE < 5) with less residues than those that are a part of a domain. Conversely, residues found in a domain should have a low PAE score with about as many residues as are part of that domain. Thus, residues near the domain-linker boundary are considered part of the domain if they have a low PAE score with at least 25 residues (a domain is expected to be >30 residues long) (São-José *et al.* 2000).

### 2.2 Evaluating and comparing performance

To validate results on our dataset of 376 lysins, we compared the predictions made by SPAED (v1.0.5) to those made by the visual delineation (ground truth; GT) and to those made by the most recent, state-of-the-art model for protein domain segmentation called Chainsaw (Wells *et al.* 2024). Chainsaw is a structure-based, supervised method that uses a convolutional neural network (CNN) to estimate the probability that pairs of residues belong to the same domain. It was shown to outperform other segmentation tools such as Merizo (Lau *et al.* 2023), EguchiCNN (Eguchi and Huang 2020), UniDoc (Zhu *et al.* 2023), and SWORD2 (Cretin *et al.* 2022).

We first compared the quality of segmentations using the intersect over union (IoU) score (Appendix 2, available as supplementary data at *Bioinformatics* online) (Tai *et al.* 2005). This score is a measure of the overlap between GT and predicted domains (with a score of 1 corresponding to a perfect overlap) (Wells *et al.* 2024). We also evaluated the accuracy of the predicted boundaries using the Domain Boundary Distance (DBD) score (Tress *et al.* 2007). This score rewards a predicted boundary that is closer to the GT boundary; one point is attributed for a perfect prediction, and 1/8 point is subtracted for every residue between the predicted and GT boundary. See Appendix 3, available as supplementary data at *Bioinformatics* online for more details.

## 3 Results

We benchmarked SPAED against Chainsaw (Wells *et al.* 2024) on a dataset of 376 endolysins by comparing the predictions made by both tools to the visual delineations made by an expert based on the predicted 3D structures. The intersect over union (IoU) and domain boundary distance (DBD) scores were used as evaluation metrics. SPAED averages an IoU-score of 96% ± 8% (SD) compared to 94% ± 7% for Chainsaw (Fig. 1B). A larger difference is observed with the DBD-score where SPAED achieves an average score of 89% ± 15% and Chainsaw has a score of 70% ± 14%.

The DBD-score measures both the accuracy (proportion of predicted boundaries that are correct) and sensitivity [proportion of ground truth (GT) boundaries that are correctly predicted] of predictions (Tress *et al.* 2007). As demonstrated in Appendix 3, available as supplementary data at *Bioinformatics* online, viewing both metrics separately allows to evaluate if a model tends to over-predict (bad accuracy; predicted linkers are longer than GT) or under-predict boundaries (bad sensitivity; predicted linkers are shorter than GT). Having both a good accuracy and sensitivity implies the predicted boundary matches the GT boundary. Figure 1B shows the accuracy and sensitivity of boundary predictions, calculated over a permissibility range that allows for distances of 0 to 7 residues between the predicted and GT boundary when classifying a prediction as correct/incorrect. SPAED tends to have a better accuracy than sensitivity, meaning that it tends to predict shorter linkers than the GT, while the opposite is true of Chainsaw. Looking at accuracy, >65% of predicted domain boundaries are predicted exactly by SPAED (Fig. 1B; accuracy at distance = 0) and, given a buffer of 2–4 residues, nearly all predicted boundaries are accurate. A remarkable 50% drop in accuracy is observed when comparing Chainsaw to SPAED for exact predictions (Fig. 1B; accuracy at distance = 0), but this difference is reduced when tolerating predictions up to 7 residues off. Regarding sensitivity, Chainsaw is better than SPAED at a distance of 0 to 3 residues, implying the GT boundary is found within the linker predicted by Chainsaw. When allowing a 4–5 residue buffer, SPAED becomes marginally better using this metric as well. Importantly, the difference in accuracy between SPAED and Chainsaw (where SPAED outperforms Chainsaw) is much bigger than their difference in sensitivity (where Chainsaw is better). Given that various parameters (PAE score, maximum number of clusters, etc.) were optimized for SPAED on the same endolysin dataset, a slight bias in the reported metrics may be observed for our tool. Conversely, Chainsaw was trained on a much greater diversity of proteins, making it, by design, less well adapted to endolysins.

The accuracy of SPAED predictions can be seen in Fig. 1C for 6 endolysins representative of different domain architectures. The examples also highlight some minor flaws in Chainsaw’s predictions, such as repeated domains that are often ignored by Chainsaw (A0A4D6AAM3, A0A1P8VVS3, A0A4D5ZYC2). In addition, SPAED can identify disordered regions, potentially signal peptides, in N- or C-termini (Fig. 1C, colored in red). These can sometimes be recovered from Chainsaw predictions if the regions were not assigned to any domain, but they are generally ignored by the tool. Finally, SPAED parameters can be adjusted, as described in the GitHub documentation, to detect terminal disordered regions and linkers more sensitively, or to optimize the algorithm for other types of proteins of interest.

Although SPAED was built for endolysins, it can also be used on other types of proteins. As a test, 18 modular proteins were collected from the CASP12 experiment (Moult *et al.* 2018, Zhou *et al.* 2019). Their 3D structures and domains were predicted using AlphaFold3 and SPAED, respectively. These, as well as the GT delineations obtained from the CASP web portal (https://predictioncenter.org/casp12/domains_summary.cgi), can be found in Fig. A4, available as supplementary data at *Bioinformatics* online. Note that SPAED parameters are tunable and were adjusted for some of these proteins as specified in the figure. For 12 proteins (A-F, L-Q), the delineations obtained by SPAED are accurate. Errors in the remaining 6 proteins result from tightly packed domains (H, J, K) or discontinuous domains (i.e. formed from two or more segments from separate regions of the protein sequence; G, I, R) that complicate the detection of boundaries in the PAE matrix. This signals a limitation of SPAED outside of endolysins that should be taken into account, especially since a relatively high proportion of domains (15%–18%) in existing databases (CATH3.5, PDB) are discontinuous (Xue *et al.* 2015). SPAED was also applied to two cellulosome components (a docking enzyme and a scaffoldin, Fig. A4 panels S, T, available as supplementary data at *Bioinformatics* online) and good delineations were obtained (Vanderstraeten *et al.* 2022). Like endolysins, their domains are compact and separate from one another, making SPAED well-suited for their accurate delineation.

To conclude, SPAED allows for the high-throughput segmentation of protein domains in a simple and interpretable manner. It is also flexible, its parameters being modifiable to more sensitively detect linkers or terminal disordered regions, or to improve segmentations if needed (e.g. for lower throughput experiments). Users can provide a folder of PAE files to annotate multiple proteins simultaneously, and it is possible to get a 3D visualization of the predicted domains by adding the protein structure files on a user-friendly website (www.spaed.ca), making it accessible to users less familiar with bioinformatics. Although it was initially developed for and optimized on endolysins, SPAED can be used on other types of modular proteins characterized by compact and relatively distant domains.

## Supplementary Material

btaf531_Supplementary_Data

## Data Availability

The data used to test SPAED can be found at https://doi.org/10.5281/zenodo.15285860.
