# Supplementary material for: SPAED: harnessing AlphaFold output for accurate segmentation of phage endolysin domains
Source: Bioinformatics. 2025 Sep 24;41(10):btaf531. doi: 10.1093/bioinformatics/btaf531 (PMC12518921; doi:10.1093/bioinformatics/btaf531)
Supplement: btaf531_Supplementary_Data [file btaf531_supplementary_data.pdf]

Supplementary material

Appendix 1: Complete example of the SPAED algorithm.

Here is the example endolysin we will be working with.

Supplementary figure A1.1:

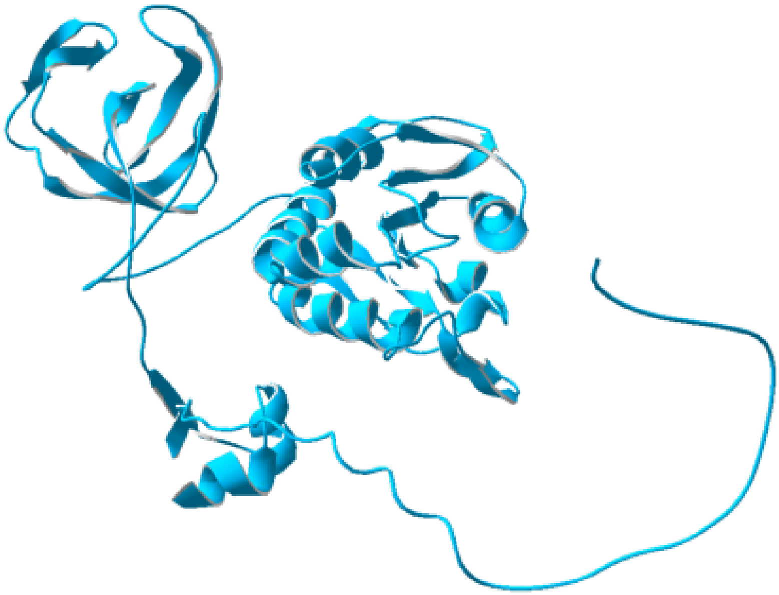

Preprocessing

SPAED takes as input the **PAE matrix** obtained from AlphaFold.

The matrix is normalized to make it symmetric:

$pae_{norm} = \frac{(pae+pae^T)}{2}$

Supplementary figure A1.2:

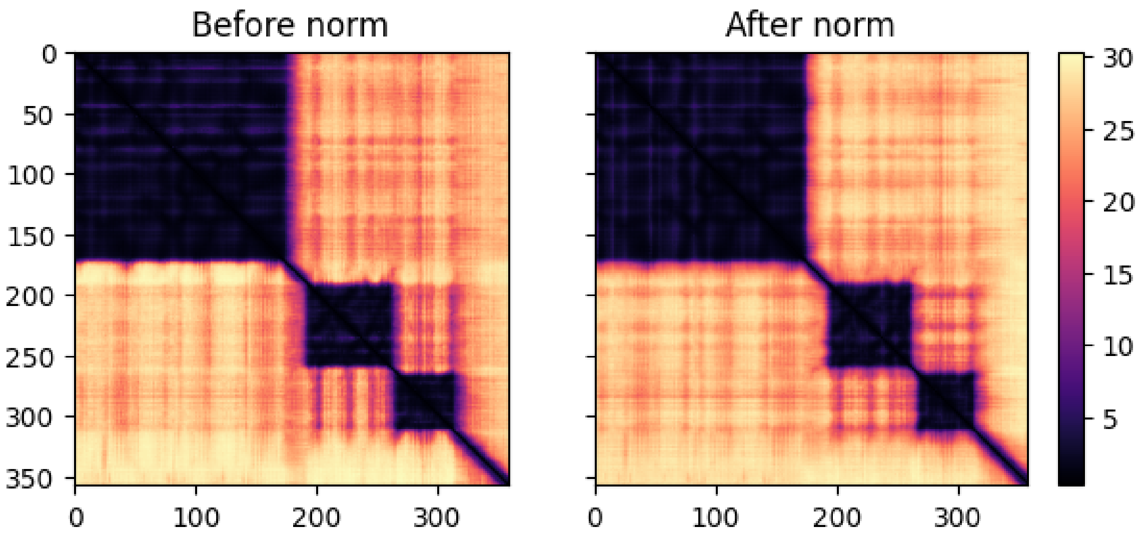

Step 1: Hierarchical clustering

Hierarchical clustering (scipy.cluster.hierarchy.fclusterdata) is performed on the normalized matrix. The maximum number of clusters is set to 1/10th of the length of the protein, so in this case to 35 clusters. This step places the most similar columns of the PAE matrix into the same clusters.

The article refers to the **profile** of a residue in the PAE matrix. The profile of a residue is obtained by taking all the values in its corresponding column of the PAE matrix. Residues in the same domain should have similar profiles. Also, notice that within a domain, residues mostly have a PAE score <=5.

Supplementary figure A1.3:

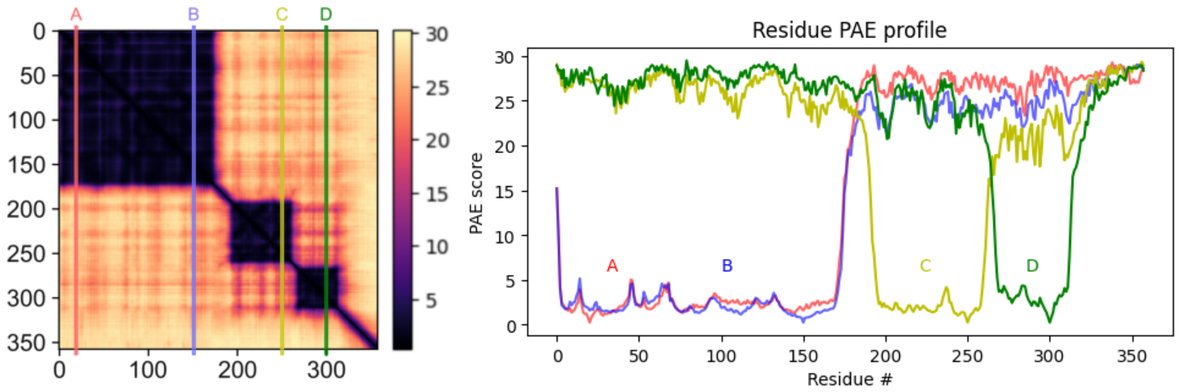

Applying the clustering algorithm yields the following result, where each colour corresponds to a different assigned cluster.

Supplementary figure A1.4:

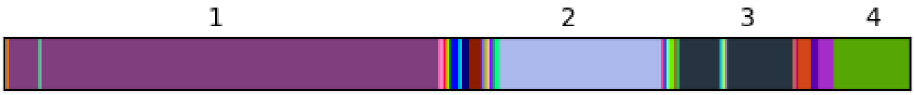

At first glance, we can already see 4 clusters, each containing many residues.

Notice how residues in linker regions are assigned to many small clusters. This is a result of the high number of clusters (in this case 35) that the clustering algorithm is allowed to look for. In contrast, long, continuous clusters are assigned to more homogeneous regions of the PAE matrix.

Step 2: Domain detection

Clusters with 25 or more residues are assigned as domains. This threshold was chosen as domains are expected to have at least 30 residues. A little buffer is given for errors present in the preliminary assignment of clusters.

Each identified domain is assigned a new cluster number (1-4 in this case) whereas all other residues are assigned a 'non-domain' id.

Supplementary figure A1.5:

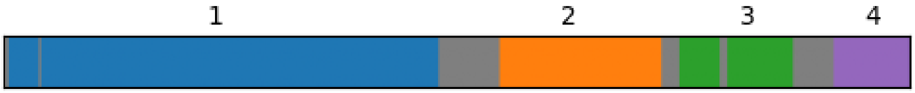

Step 3: N- and C-terminal correction

Next, we correct the ends of the sequences. These regions often possess residues that are freer to move and thus have a profile in the PAE matrix that is very different from residues in the domain they are nearest to. If this region is very short (<20 residues), it is simply concatenated to the nearest domain (like the first residue in this example).

Endolysins can also possess signal peptides in N- or C-terminal which correspond to longer (~25 residues) disordered regions. These are interesting to flag for future studies. In the PAE matrix, these less packed (disordered) regions can be identified by looking for low PAE score 'diagonals'. In practice, we can count the number of 'dark' squares (PAE score <5) in every column (see figure 3 for the reason for using PAE score <5). This gives a good estimation of the packing of the protein in any given region as it reflects the number of residues that are close in space to any given residue. More importantly, residues in less packed regions such as linkers or end-terminal disordered regions will have very few of these 'dark' squares (i.e. they correspond to the 'diagonal' regions).

Supplementary figure A1.6:

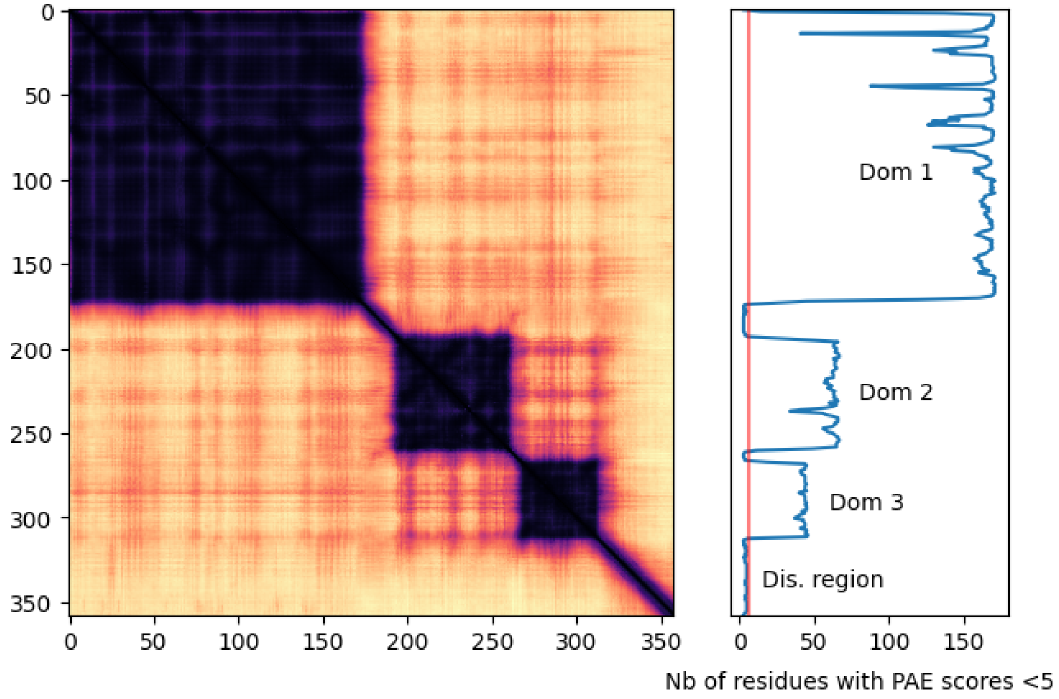

If more than 80% of residues in a region are below the cutoff (red line in the graph above) corresponding to 6 residues with scores < 5, the region is considered to be disordered.

The new assignment yields the following, where the 4th region, now in red, is flagged as being disordered.

Supplementary figure A1.7:

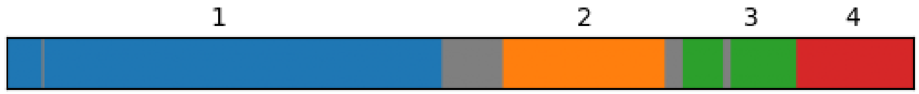

Step 4: Artifact removal

We correct small artifacts originating from the initial assignment of clusters by scanning for residues assigned as ‘non-domain’ within a domain. This fixes the wrongly assigned residues like in domain 3.

Supplementary figure A1.8:

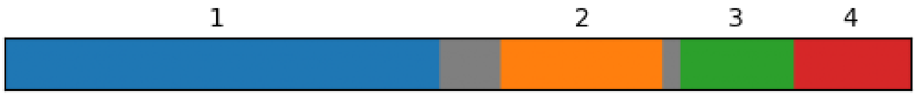

Step 5: Linker adjustment

Finally, we adjust the length of linkers. This step is necessary to correct small mistakes at the domain/linker interface that can originate from the initial clustering.

Residues near the domain/linker boundary are considered part of the domain if they have a low PAE score (<5) with at least 25 residues (a domain is expected to be at least 30 residues long) as residues found in a domain should have a low PAE score with about as many residues as are part of that domain.

Supplementary figure A1.9:

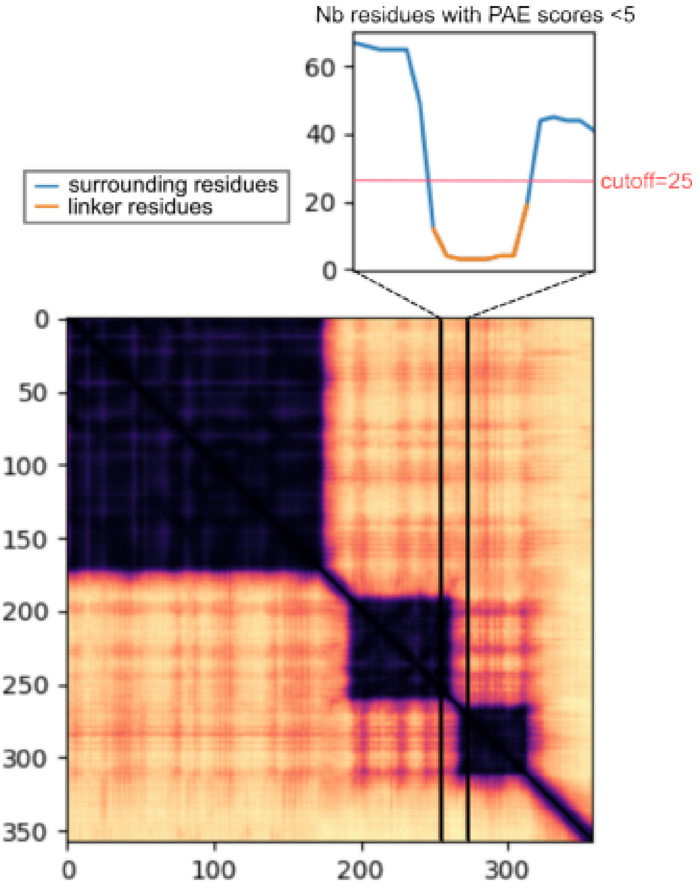

Supplementary figure A1.10:

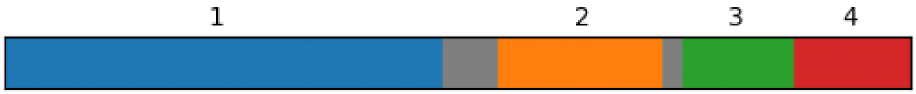

Final result

Finally, we can see how the predicted domains look on the predicted 3D structure.

Supplementary figure A1.11:

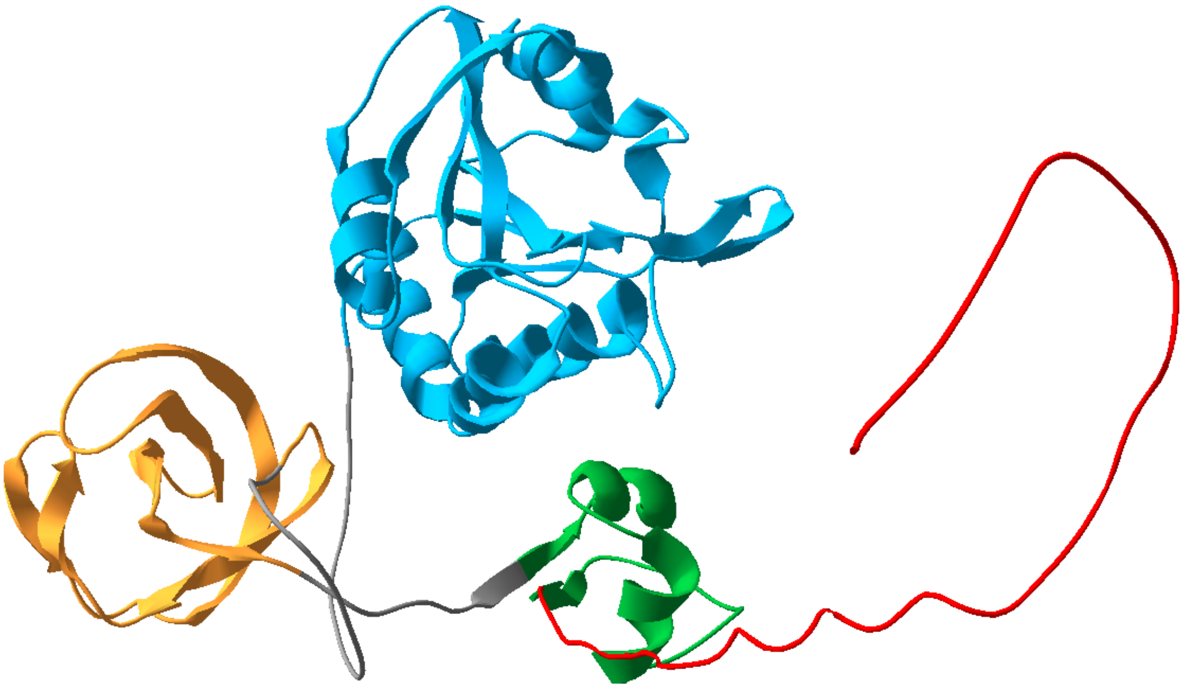

Appendix 2: Additional explanation of Intersect over Union (IoU) score.

As described in Wells et al. (Wells 2024): following the approach of Merizo (Lau 2023), for a given protein chain we compute the average intersection over union (IoU) between paired sets of predicted and ground-truth residues assigned to each domain. As a first step, each ground-truth domain is paired with a predicted domain such that the sum of all intersections over unions is maximised while respecting the following constraints: Each ground-truth domain (represented as a set of residue indices) Ti can have, at most, one paired predicted domain Pi. Second, each predicted domain can be assigned at most once. No IoU is computed for the sets of residues that are labelled as, or predicted to be non-domain residues. n_{dom} corresponds to the number of ground-truth domains. To generate a final score for the whole chain, each domain-level IoU is weighted by the number of residues in the ground-truth domain:

$$\text{IoU} = \sum_{i=1}^{n_{dom}} \frac{|T_i \bigcap P_i|}{|T_i \bigcup P_i|} \cdot \frac{|T_i|}{\sum_{j=1}^{n_{dom}} |T_j|}.$$

Appendix 3: Additional explanation for domain boundary evaluation

Domain boundary score (Tress 2007): rewards a predicted boundary that is closer to the Ground Truth (GT) boundary. One point is attributed for a perfect prediction and 1/8 point is subtracted for every residue between the predicted and GT boundary. If the domain boundary has a linker, the whole linker is regarded as the domain boundary.

Accuracy of domain boundaries: proportion of correctly predicted boundaries.

Sensitivity of domain boundaries: proportion of GT boundaries predicted correctly.

Accuracy and sensitivity are calculated over a permissibility range (e.g. the accuracy considering that predictions within a certain number of residues of the GT are correct). This is because domain boundary predictions are, more often than not, imperfect. Allowing a buffer of a few residues allows for a better understanding of the model's performance.

Let us look calculate the accuracy and sensitivity for the following example, **considering a permissibility range (distance) of 1 residue**:

Supplementary figure A3:

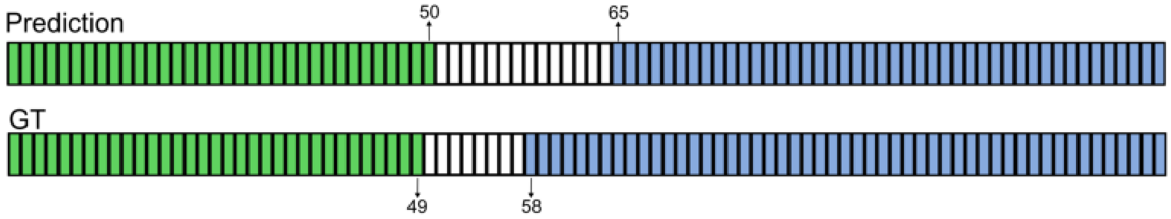

Accuracy: The predicted boundary of the green domain (residue 50) is found within the GT linker (residues 50-57). It is considered to be correct. The boundary of the blue domain (residue 65) is 7 residues away from the GT boundary (residue 58). It is counted as an error. The accuracy is the proportion of correctly predicted boundaries: 1 good prediction / 2 predictions = 50%.

Sensitivity: The GT boundary of the green domain (residue 49) is 1 residue away from the predicted boundary (residue 50). At a permissibility range of 1 residue, it is considered to be correct. The GT boundary of the blue domain (residue 58) is found within the predicted linker (residues 51-64). It is also considered to be correct. The sensitivity is the proportion of GT boundaries predicted correctly: 2 correct GT boundaries / 2 GT boundaries = 100%.

Cases where a model predicts linkers that are longer than the GT will result in a good sensitivity but a bad accuracy because the predicted boundary will have a tendency to be found outside the GT linker. Conversely, cases where a model predicts linkers that are shorter than the GT will result in a good accuracy but a bad sensitivity because the predicted boundary will have a tendency to be found within the the GT linker.

To generate the accuracy and sensitivity curves in Figure 2B, the accuracy and sensitivity were calculated allowing for a buffer (distance) of 0-7 residues.

Appendix 4: Example segmentations of CASP12 proteins and cellulosomes.

Supplementary figure A4: Comparison between domain delineations obtained with SPAED and the ground truth (GT) for proteins from the CASP12 experiment (**A-R**), as well as two example cellulosome components (**S, T**). SPAED was launched with default parameters unless specified otherwise. Segments in red correspond to disordered regions. In SPAED predictions, segments in grey correspond to linkers; in GT structures, they correspond to undefined regions.

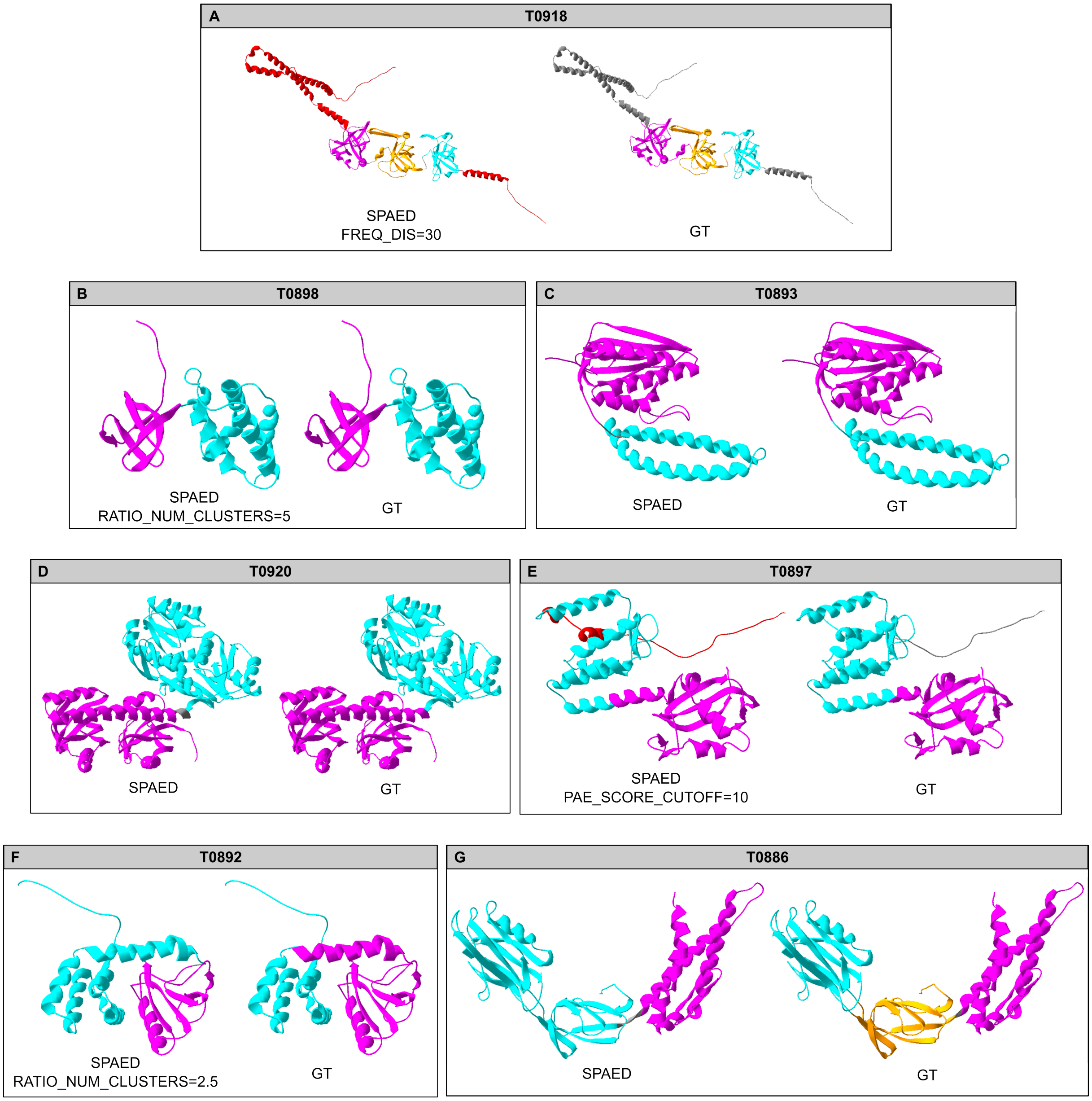

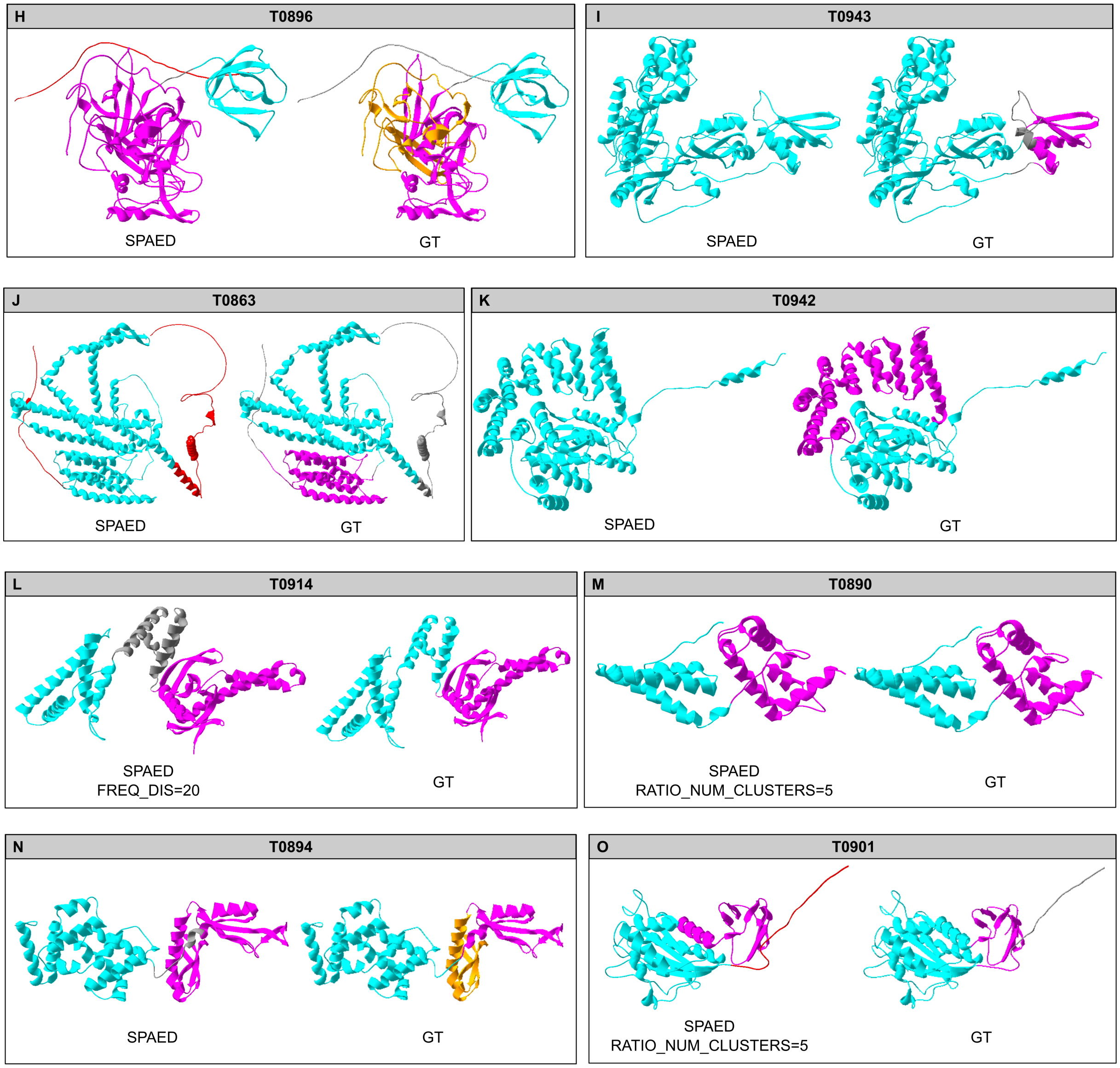
H
T0896
SPAED
GT
I
T0943
SPAED
GT
J
T0863
SPAED
GT
K
T0942
SPAED
GT
L
T0914
SPAED
FREQ_DIS=20
GT
M
T0890
SPAED
RATIO_NUM_CLUSTERS=5
GT
N
T0894
SPAED
GT
O
T0901
SPAED
RATIO_NUM_CLUSTERS=5
GT

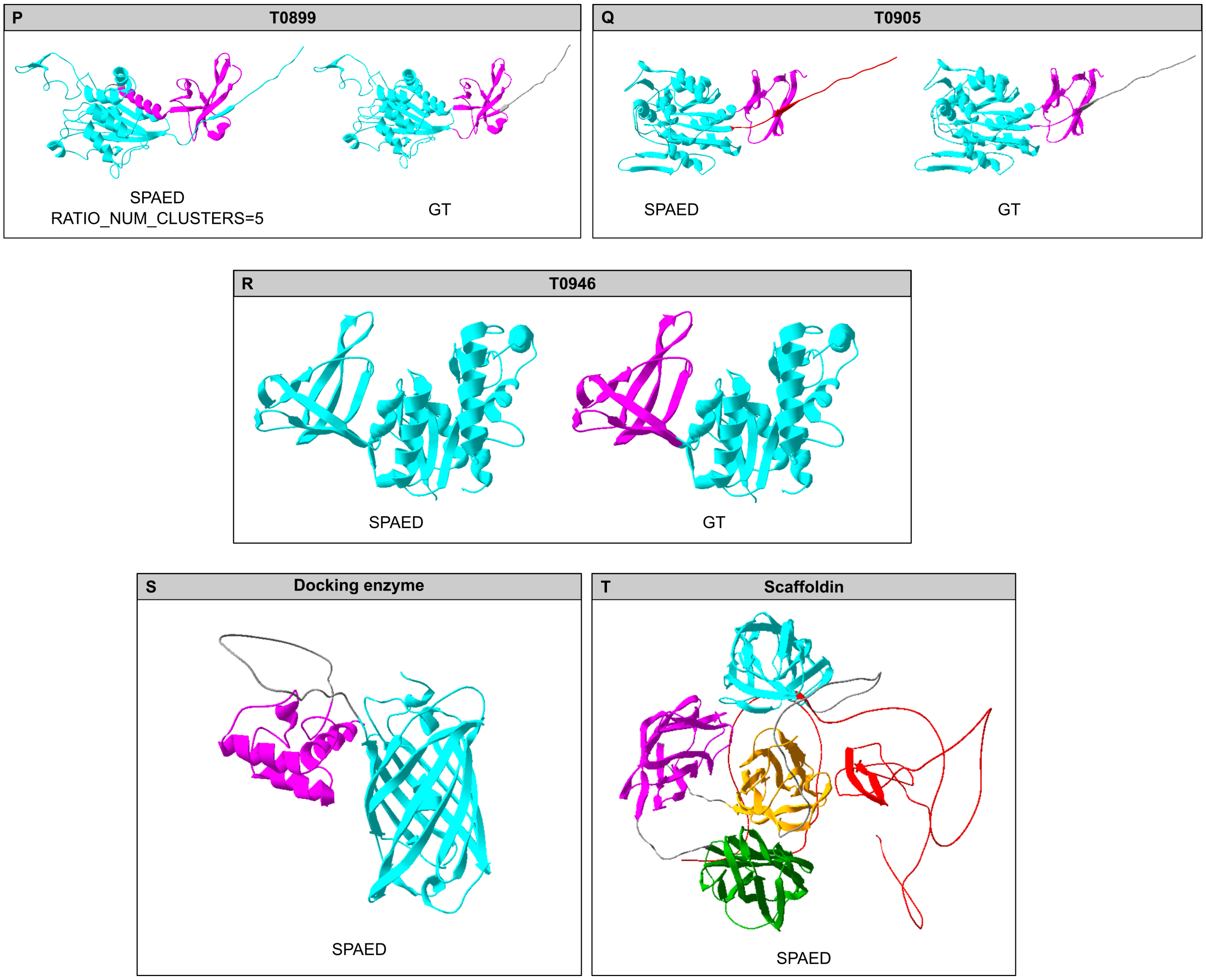
P
T0899
SPAED
RATIO_NUM_CLUSTERS=5
GT
Q
T0905
SPAED
GT
R
T0946
SPAED
GT
S
Docking enzyme
SPAED
T
Scaffoldin
SPAED
